# Dynamic Constitutive Model of Basalt Fiber Concrete After High Temperature Based on Fractional Calculus

**DOI:** 10.3390/ma18204657

**Published:** 2025-10-10

**Authors:** Wenbiao Liang, Kai Ding, Yan Li, Yue Zhai, Lintao Li, Yi Tian

**Affiliations:** 1 School of Science, Chang’an University, Xi’an 710064, China; lwb23@chd.edu.cn; 2 School of Geological Engineering and Geodesy, Chang’an University, Xi’an 710064, China; liyanlwbdlp@chd.edu.cn (Y.L.); zy@chd.edu.cn (Y.Z.); lilintaoxk@163.com (L.L.); 2023226154@chd.edu.cn (Y.T.)

**Keywords:** BFRC, dynamic constitutive model, fractional calculus, viscoelasticity, high-temperature damage

## Abstract

Concrete materials undergo a series of physical and chemical changes under high temperature, leading to the degradation of mechanical properties. This study investigates basalt fiber-reinforced concrete (BFRC) through high-temperature testing using the split Hopkinson pressure bar (SHPB) apparatus. Impact compression tests were conducted on specimens after exposure to elevated temperatures to analyze the effects of varying fiber content, temperature levels, and impact rates on the mechanical behaviors of BFRC. Based on fractional calculus theory, a dynamic constitutive equation was established to characterize the viscoelastic properties and high-temperature damage of BFRC. The results indicate that the dynamic compressive strength of BFRC decreases significantly with increasing temperature but increases gradually with higher impact rates, demonstrating fiber-toughening effects, thermal degradation effects, and strain rate strengthening effects. The proposed constitutive model aligns well with the experimental data, effectively capturing the dynamic mechanical behaviors of BFRC after high-temperature exposure, including its transitional mechanical characteristics across elastic, viscoelastic, and viscous states. The viscoelastic behaviors of BFRC are fundamentally attributed to the synergistic response of its multi-phase composite system across different scales. Basalt fibers enhance the material’s elastic properties by improving the stress transfer mechanism, while high-temperature exposure amplifies its viscous characteristics through microstructural deterioration, chemical transformations, and associated thermal damage.

## 1. Introduction

Concrete is the most widely used material in engineering construction, and the generation and development of internal cracks during service is the fundamental cause of its failure [1]. Studies have shown that the incorporation of basalt fibers into concrete can effectively inhibit the initiation and development of internal cracks, thereby enhancing the strength, toughness, high-temperature resistance, and corrosion resistance of the material [2,3,4,5,6,7,8]. With the frequent occurrence of earthquakes, fires, and terrorist attacks, BFRC has been gradually applied to major foundation projects threatened by extreme loads such as high temperature, explosion, and shock, such as in nuclear reactor engineering and deep underground engineering, and its dynamic mechanical properties have also been studied [8,9,10,11,12]. After being subjected to high temperature, a series of physical and chemical changes will occur in fiber concrete, resulting in loose internal structure [13], resulting in a material “softening” phenomenon characterized by the decrease in elastic modulus, the decrease in compressive strength, and the increase in peak strain [14]. At the same time, the strain rate effect will improve the performance of concrete, and the interaction of the two influences makes the mechanical behavior complex and difficult to predict. Therefore, for BFRC materials that have been used in high-temperature environments for a long time or after sudden fire, it is very important to establish an accurate rate–temperature constitutive relationship and reveal the evolution of material mechanical properties for structural safety design and reliability assessment.

The constitutive model is an important basis for the structural design, safety performance analysis, and life prediction of concrete. At present, regarding the construction of a constitutive equation of concrete material considering temperature and strain rate effect, some scholars start from the angle of elasticity or elastic–plastic analysis. For example, Jia et al. introduced the damage parameter, temperature weakening effect coefficient, and strain rate strengthening factor based on the classical Hooke’s law and established the high-temperature dynamic constitutive equation of concrete [15]. Based on a static nonlinear elastic constitutive model, Wang Zhikun et al. [16] introduced damage variables and strain rate strengthening factors based on concrete wave impedance to construct a dynamic damage constitutive model of geopolymer concrete at high temperature. Wang Hongwei et al. [17] and Ren et al. [18] have similar research methods. The Sargin nonlinear elastic model under general working conditions was extended to the high-temperature dynamic working condition. Kou et al. [19] and Yu et al. [20] both modified the high-temperature dynamic damage constitutive relationship based on the DP criterion. For example, Wang Liwen et al. [21], based on the ZWT nonlinear viscoelastic constitutive model, introduced the function of strain and strain rate as the damage factor and obtained the ZWT constitutive model suitable for high-temperature dynamic working conditions. Based on a nonlinear viscoelastic element, Wang Fei et al. [22] introduced the function of strain and strain rate as the damage factor to construct the constitutive relationship. At present, most of the research on the rate and temperature constitutive of concrete is based on the perspective of elasticity and elastoplastic, but there are few concrete constitutive models based on viscoelasticity that consider both temperature and strain rate.

If the viscoelasticity of concrete is ignored, the calculation can be simplified to a certain extent. However, concrete is a composite material containing aggregate, pores, and various defects (such as grain boundaries, cracks, and holes); these characteristics lead to the reflection and diffraction of stress waves. Therefore, it is necessary to establish a constitutive model considering the viscoelasticity of concrete to accurately predict the performance of materials under complex loading conditions and theoretically elucidate the mechanism of high-temperature deterioration of concrete materials [23].

Fractional calculus theory is a mathematical theory to study the characteristics of differential and integral operators of arbitrary order. The physical model established based on fractional calculus can describe the physical phenomena with memory and time dependence well [24], and it can be used to study the viscoelastic characteristics of materials [25], which can accurately describe the mechanical behavior of materials with fewer parameters. Researchers [26,27,28,29] have already employed fractional calculus to model the mechanical behavior of concrete. Models based on fractional calculus can accurately capture the time-dependent behavior of concrete while requiring fewer parameters and maintaining a relatively concise constitutive relationship.

This study conducts high-temperature heating experiments on BFRC and performs impact compression mechanical tests using the SHPB system. The objective is to develop a unified model framework based on fractional calculus theory, integrating high-temperature damage, strain rate effects, and viscoelastic characteristics. A high-temperature dynamic damage constitutive model for BFRC is established, capable of simultaneously reflecting high-temperature damage, strain rate effects, and viscoelastic behavior. Additionally, scanning electron microscopy and nuclear magnetic resonance experiments are employed to reveal the viscoelastic evolution mechanisms of BFRC under high-temperature conditions, providing microscopic physical support for the model.

## 2. BFRC High-Temperature Tests

### 2.1. Specimens Preparation

The strength grade of the concrete matrix is C35: cement(P.O.42.5, Shaanxi Qinling Cement Building Materials Co., Ltd., Shaanxi, China)/sand/stone/water/fly ash/water reducing agent = 1:2.03:3.80:0.56:0.40:0.02. P.O.42.5 Portland cement is used for cement, continuous graded medium sand with a fineness modulus of 2.42 is used for fine aggregate, and 5–12 mm continuous graded gravel is used for coarse aggregate. The grade of fly ash is Ⅱ, fineness is 43 μm, density is 2.4 g/cm^3^, and moisture content is about 5%. The water reducing agent uses FDN naphthalene series high-efficiency water reducing agent, with a PH range between 7 and 9 and a water reduction rate of about 20–30%. The basalt fiber has diameter 17.4 μm, length 12 mm, tensile strength 2000 MPa, elastic modulus 85 GPa, elongation 2.5%, density 2699 kg/m^3^, respectively, according to the volumes of 0.1%, 0.2%, 0.3%, and 0.4% mixed into the concrete matrix. Tap water was used for the test. The mix proportions of the test specimens are presented in Table 1.

The preparation process of the specimens was carried out in accordance with the relevant provisions of the Technical Regulations for the Application of Fiber Concrete (JGJ/T221-2010) [30]; following the preparation of the test blocks, cores were drilled, sectioned, and polished to produce the specimens. Firstly, the raw materials (except water) weighed according to the ratio were fully dry mixed with a blender (HJW-60, Wuxi Jianyi Instrument & Machinery Co., Ltd., Wuxi, China), so that the fibers were evenly dispersed in the aggregate, and then wet mixed with water. Then, the flowing concrete was poured into cube molds with a side length of 150 mm, and the molds were placed on a shaking table for 30 s. The molds were left standing at room temperature for 24 h until they were solidified and formed. Then, they were placed in the standard constant temperature and humidity curing box (YH-40B, Cangzhou Blue Beauty Instrument Co., Ltd., Cangzhou, China) according to the standard curing conditions for 28 days. Finally, the test blocks were drilled, cut, and polished into a cylinder of dynamic test specimens with a diameter of 48 mm and height of 25 mm.

### 2.2. Research Program

To systematically investigate the effects of high-temperature cooling on the dynamic mechanical properties of BFRC, a series of experiments were designed and conducted. After preparing the BFRC specimens, they were subjected to five temperature gradients under high-temperature water cooling. To eliminate the influence of moisture content, the specimens cooled in water were air-cured for four weeks before undergoing impact tests at three different impact velocities. Each test condition was repeated three times, resulting in a total of 225 specimens. The experiments yielded the strain rate and stress–strain curves of BFRC under various conditions, which are intended for theoretical analysis. The temperature gradients and impact velocities are detailed in the Table 2 provided.

### 2.3. High-Temperature Test and High-Temperature Damage Characteristics

**Figure 1:**
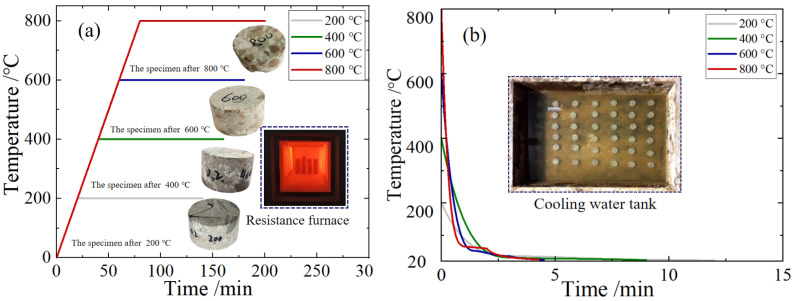
Specimen preparation and high-temperature cooling test: (**a**) heating curve; (**b**) cooling curve.

The processed BFRC specimens were put into the intelligent box resistance furnace (SX2-8-12TP, Shanghai Yiheng Scientific Instrument Co., Ltd., Shanghai, China) for the high-temperature heating test. The heating rate was set as 10 °C /min, and the target temperatures were 20 °C, 200 °C, 400 °C, 600 °C, and 800 °C, respectively. After reaching the target temperature, the constant temperature was maintained for 2 h to ensure that the specimens were fully heated [31]. The heating curves under different temperature conditions and the high-temperature damage characteristics of the specimen are shown in Figure 1a. The specimens were subsequently removed from the electric resistance furnace and subjected to water bath cooling in a tank measuring 1 m × 0.8 m × 0.4 m. Thermometer probes were placed on the specimen surfaces to monitor the temperature variation, as shown in Figure 1b. After the temperature of the specimens dropped to normal temperature, they were placed in a dry and ventilated place for 2 weeks to achieve equilibrium moisture content and to prepare for subsequent impact mechanical tests.

As can be seen from Figure 1, when the temperature reached 200 °C, the appearance of the specimens was relatively complete, the surface color was bluish gray, and no crack or skin peeling was observed; the sound was deep and resonant when they were hit. When heated to 400 °C, the appearance of the specimens was still complete, but the surface gray was aggravated, and there was still no crack or skin peeling phenomenon. When they were knocked, the sound was clearer than at 200 °C. When they were cooled in the water tank, white mist was produced, and blisters appeared on the surface of the specimens. When heated to 600 °C, the surface of the specimens was gray, with slight cracks on the surface accompanied by slight peeling of the skin, and the knocking sound was clear. When cooled in the water tank, a large amount of white mist and water splash was generated. When they were heated to 800 °C, the surface of the specimens was gray and slightly red; there were slight cracks and an obvious skin peeling phenomenon. When they were cooled in the water tank, a large amount of white mist was produced, the splash intensified, and white suspended objects appeared in the water. After cooling of the specimens, the water in the box was turbidified, and some specimens were seriously incomplete.

The relevant research [32,33,34] findings indicate that when concrete is exposed to elevated temperatures, particularly above 500 °C, an excessive cooling rate can induce a thermal shock effect. This phenomenon arises from the rapid cooling and contraction of the concrete surface while the interior remains at a high temperature and in an expanded state, leading to uneven thermal stresses and the formation of microcracks. Additionally, the evaporation of internal moisture into steam at high temperatures, followed by rapid condensation during water cooling, creates localized vacuum and pressure fluctuations, further exacerbating cracking. Studies have identified a critical cooling rate threshold of 44 °C/min for the onset of thermal shock. In this experiment, the minimum cooling rate employed was 93 °C/min, which unequivocally meets the conditions for thermal shock. Furthermore, observable damage was noted in specimens subjected to water cooling at 600 °C and 800 °C, consistent with the manifestations of thermal shock. Therefore, the phenomena observed in the high-temperature water-cooling tests can be attributed to the thermal shock effect.

## 3. Impact Compression Tests and Factors Analysis

### 3.1. Impact Compression Tests of BFRC Specimens

**Figure 2:**
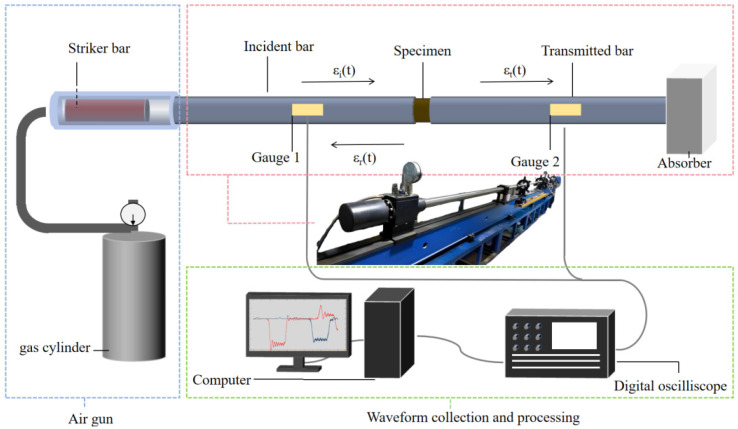
SHPB test device.

The SHPB system is currently the most representative and relatively accurate test device for studying the impact mechanical characteristics of brittle materials [35]. In this paper, a Φ50 mm SHPB device (WAW3000A, Hengle Xingke Instruments, Jinan, China) (as shown in Figure 2) was used in the Explosion and Impact Protection Laboratory of Chang‘an University to carry out impact compression tests on BFRC specimens with different volume content and different temperature conditions. The impact rates were set as 5.4 m/s, 8.8 m/s, and 11.3 m/s, respectively, and the “two-wave method” was used to calculate the stress [36]. Upon acquiring the raw data, waveform distortions and datasets failing to reach pressure equilibrium were excluded. Only data with reliable waveforms and stresses closest to complete equilibrium were retained as the subject of study to ensure the validity of the results [37,38].

### 3.2. Mechanical Properties and Factors Analysis

(1) Fiber content

**Figure 3:**
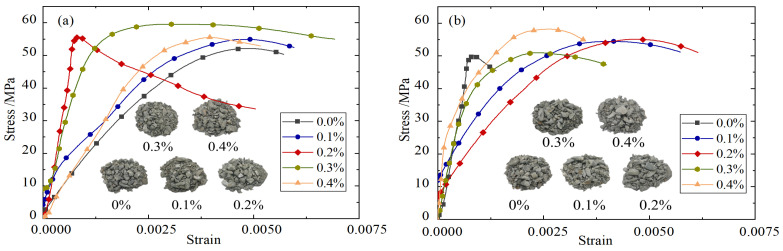

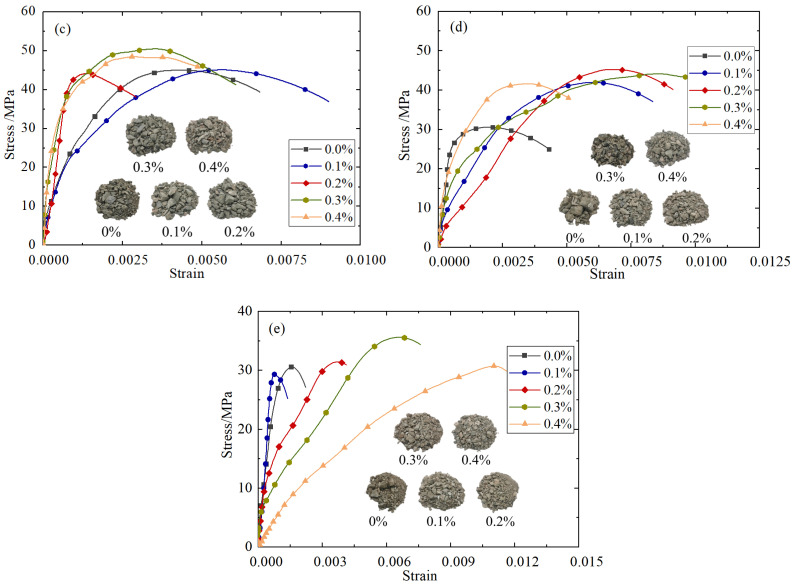
Stress–strain curves of BFRC with different fiber content: (**a**) 20 °C; (**b**) 200 °C; (**c**) 400 °C; (**d**) 600 °C; (**e**) 800 °C.

**Figure 4:**
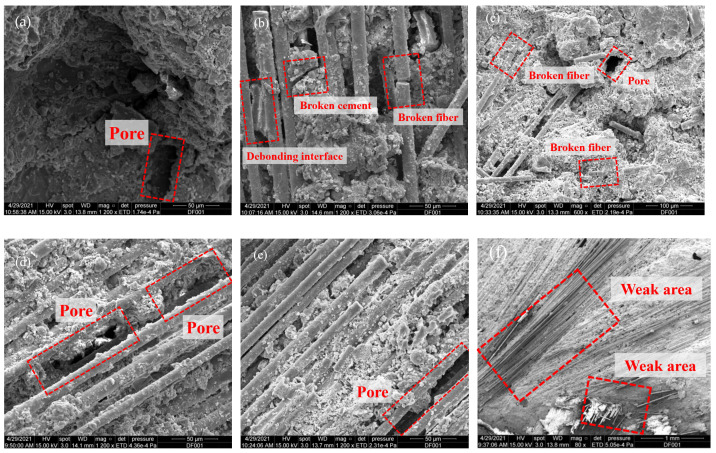
SEM microscopic topography of specimens with different fiber content: (**a**) 0%; (**b**) 0.1%, (**c**) 0.2%; (**d**) 0.3%; (**e**) 0.4%; (**f**) weak area.

Taking the impact rate of 8.8 m/s as an example, the stress–strain curves of BFRC under different fiber content conditions are shown in Figure 3. In order to further reveal the working mechanism of basalt fiber and its influence on the mechanical properties of the sample under impact compression, Electron microscope scanner (Quanta 650, FEI Company, Hillsboro, OR, USA) was used to carry out electron microscopy tests, and the microscopic morphology of BFRC with different fiber content was obtained, as shown in Figure 4.

It can be seen from Figure 3 that the strength and toughness of BFRC specimens are basically better than those of plain concrete specimens after high temperatures of 600 °C and 800 °C. The reason for this is that when the BFRC specimens crack, the fiber cemented with the mortar appears to debond and slip, which plays a bridging role. For example, as shown in Figure 4b, cracks appear in the concrete marked by the red box in the middle, and the stress of the concrete is transferred and borne by the surrounding basalt fibers through the bridging action after release. The cylindrical concave mortar block marked by the red box on the left is obviously the mortar area separated from the fiber debonding during the loading process. In the process of the loading and cracking of the BFRC specimen, the stress generated by the fiber is transmitted to the surrounding uncracked matrix. When the stress transmitted by the fiber reaches the cracking strength of the surrounding matrix, a new crack is formed in the matrix, and so on; thus the composite will undergo multiple stable cracking and show toughness enhancement behavior. However, if the fiber bears too much load after the first crack, the fiber may also be directly broken, as shown in the red box on the right of Figure 4b.

It can also be seen from Figure 3 that the amount of fiber in the concrete matrix and the dispersion effect are directly related to the macroscopic mechanical properties of basalt fiber concrete. In general, the peak stress of BFRC specimens first increases and then decreases with the increase in fiber content, and the dynamic compressive strength reaches the highest when the fiber volume content is about 0.2%. This may be due to the fact that, when the fiber admixture is too low, the bridging effect is limited, as shown in Figure 4a,b. However, when the amount of fiber is too large, the basalt fiber has a smooth surface and lacks polar hydrophilic groups such as the amino group and carboxyl group, which leads to weak interaction with water, and it is easy to form “agglomerations” in the cement paste, which cannot be fully cemented with the mortar and form void defect areas, thus affecting the compactness of the sample, as shown in Figure 4e,f.

(2) Temperature

**Figure 5:**
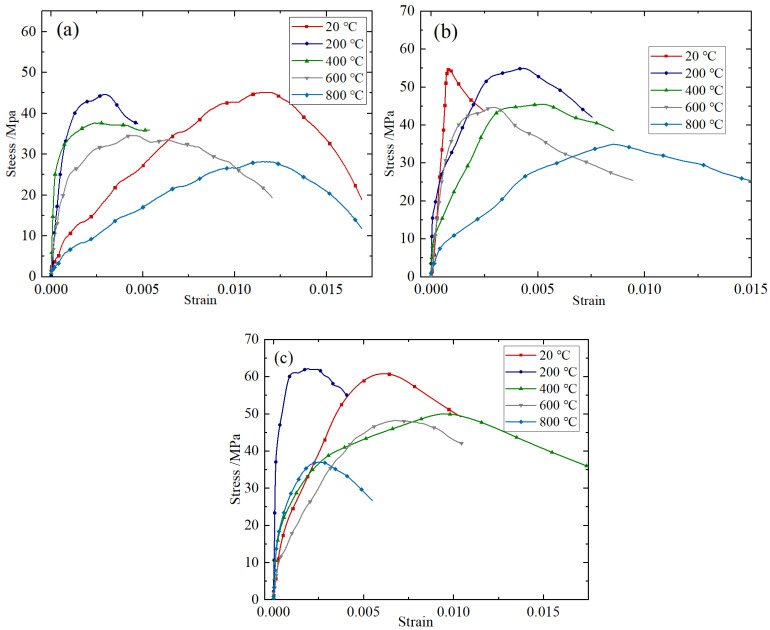
Stress–strain curves at different temperatures: (**a**) 5.4 m/s; (**b**) 8.8 m/s; (**c**) 11.3 m/s.

Taking the working condition of 0.2% fiber content as an example, the stress–strain curves of the BFRC specimens under different temperature conditions are shown in Figure 5.

It can be seen from Figure 5 that under the three impact rates, the peak stress of BFRC specimens decreases as a whole with the increase in high temperature, showing a significant high-temperature deterioration effect. BFRC will undergo a series of physical changes and chemical reactions under different temperature operating conditions. In the temperature range of 100–200 °C, the hydration products in BFRC gradually decompose [13]. When the temperature reaches 300 °C, the internal C-S-H of BFRC is destroyed [39]. After the temperature reaches 400 °C, the chemical decomposition of calcium hydroxide crystallization occurs. Limestone decomposes after 600 °C [15]. When the concrete is heated, chemical reactions continue to occur, leading to the deterioration of internal structure, and when the specimen is cooled by water, the extremely uneven temperature difference between the interior and exterior leads to the full development of internal cracks in the specimen. The higher the temperature, the greater the temperature difference between the interior and exterior, and the more serious the cooling damage caused; the slurry greatly shrinks while the aggregate is heated and expanded, the internal stress becomes larger, and the bonding surface of aggregate and slurry cracks. The internal structure of the specimen was damaged, and the compressive strength decreased sharply.

(3) Impact rate

**Figure 6:**
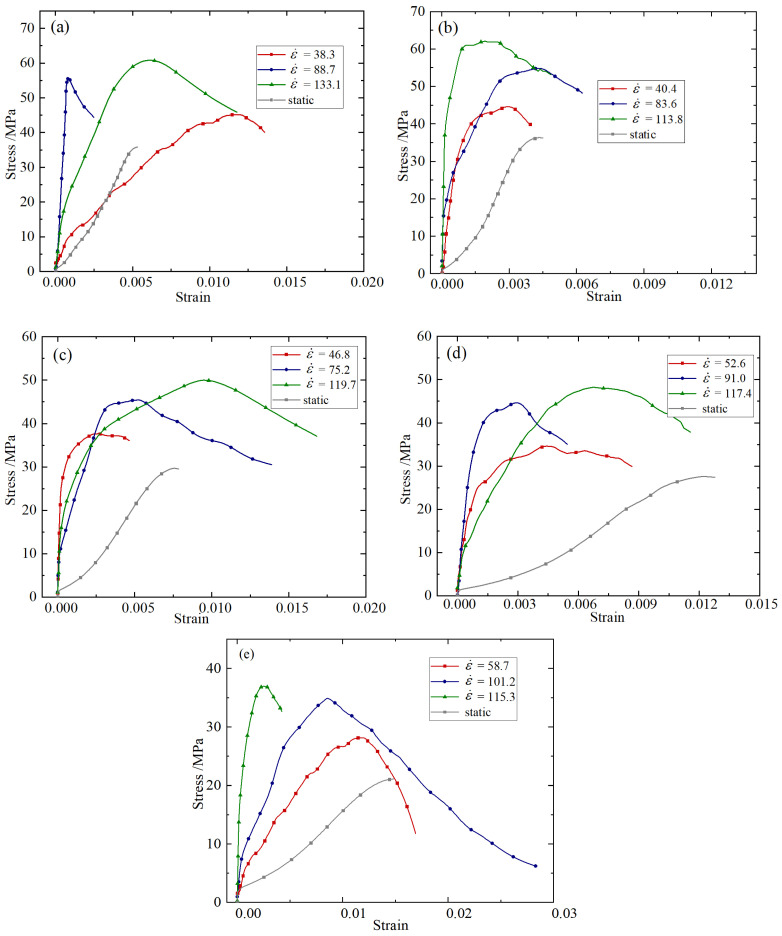
Stress–strain curves at different impact rates: (**a**) 20 °C; (**b**) 200 °C; (**c**) 400 °C; (**d**) 600 °C; (**e**) 800 °C.

Taking the working condition of fiber content 0.2% as an example, the strain rate of BFRC specimens under different impact rates under the condition of 20–800 °C was calculated, and the stress–strain curve was plotted as shown in Figure 6.

It can be seen from Figure 6 that BFRC has an obvious strain rate strengthening effect, that is, the peak stress of the specimen increases significantly with the increase in strain rate. This result is caused by the differences in the generation and development of cracks under different strain rates [40]. Compared with the energy required for crack development, the energy required for crack generation is larger. At lower strain rates, there are fewer cracks, and they are mainly generated and developed along the weak aggregate mortar interface; so the energy required for specimen failure is smaller. And some cracks develop along the mortar or even the aggregate, requiring greater energy and showing higher strength. The strain rate strengthening effect is a key factor in predicting the load capacity of building structures under extreme conditions.

In order to further quantify the strain rate strengthening effect, the expression of dynamic enhancement factor DIF is defined as [41]:

(1) $$DIF=\frac{f_{\mathrm{cd}}}{f_{\mathrm{cs}}}$$

The corresponding DIF values were calculated as shown in Table 3.

Research shows that the DIF of concrete material and the logarithm of the strain rate approximately satisfy a linear relationship [42], namely:

(2) $$DIF=a+b\mathrm{lg}\dot{\varepsilon }$$

**Figure 7:**
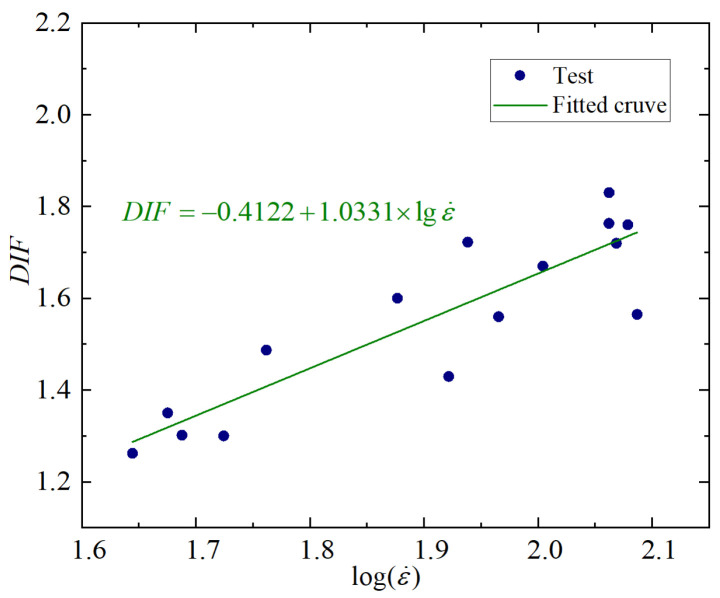
Variation rules of DIF with the logarithm of strain rate.

Based on the data of this test, fitting Equation (2) yields a = −0.4122, b = 1.0331, and goodness of fit R^2^ = 0.9382. The fitting result is good, as shown in Figure 7.

## 4. Damage Constitutive Model Based on Fractional Calculus Theory

### 4.1. Constitutive Model Construction

Fractional calculus theory is a mathematical theory that studies the characteristics of differential and integral operators of arbitrary order. It has advantages in describing physical phenomena with memory and time dependence. Based on the fractional calculus theory, the dynamic constitutive model of BFRC is established by considering the high-temperature damage, strain rate effect, and viscoelastic characteristics of the material.

The stress–strain relationship of an ideal fluid satisfies Newton’s law of viscosity, and the stress–strain relationship of an ideal solid satisfies. Considering the differential order, it can be rewritten; then the concrete material between the ideal fluid and ideal solid can be considered as [43]:

(3) $$\sigma (t)=\frac{\xi d^{\beta }\varepsilon (t)}{dt^{\beta }},\;(0\leq \beta \leq 1)$$

where ξ is a parameter similar to the elastic modulus in Hooke’s law, and β is the fractional differential order. When the order approach decreases to 0, the fractional differential degenerates into the identity operator of integer order, and the constitutive equation degenerates into the linear stress–strain relationship of the elastic body; when the order increases to 1, it is transformed into a viscous response. The equation can describe the mechanical behavior of the material in the transition state of the elastic–viscoelastic–viscous continuum through the parameters β.

In this paper, the SHPB test uses the shaper filter; so it can be regarded as a constant strain rate loading process [35]. Under constant strain rate $\varepsilon =\dot{\varepsilon }t$, $\dot{\varepsilon }$ is the average strain rate, and Equation (3) can be further deformed as follows:

(4) $$\sigma (t)=\xi \dot{\varepsilon }\frac{d^{\beta }t}{dt^{\beta }},\;(0\leq \beta \leq 1)$$

In order to solve $d^{\beta }t/dt^{\beta }$, according to the theory of Riemann–Liouville type fractional calculus operator, the β order integral of the function f(t) is as follows:

(5) d−βf(t)dt−β=Dt−βt0f(t)=∫t0t(t−τ)β−1Γ(β)f(τ)dτ

where Γ(β) is the Gamma function.

Then the fractional differential is given by

(6) dβf(t)dtβ=Dtβt0f(t)=dndtn[Dt−(n−β)t0f(t)]

For the function f(t)=ct (c is constant), it is mathematically possible to obtain an exact solution of its fractional calculus, and when 0≤β≤1, its fractional differential is given in Equation (7)

(7) $$\frac{d^{\beta }f(t)}{dt^{\beta }}=\frac{c}{\Gamma (2-\beta )}t^{1-\beta }$$

Substituting $\varepsilon =\dot{\varepsilon }t$ and Equation (7) into Equation (4) to solve the fractional calculus constitutive equation of concrete materials, the dynamic constitutive equation of fractional calculus under constant strain rate can be obtained as

(8) $$\sigma (\varepsilon )=\frac{\xi }{\Gamma (2-\beta )}{\dot{\varepsilon }}^{\beta }\varepsilon^{\left(1-\beta \right)},\;(0\leq \beta \leq 1)$$

The microstructural differences in the loading process of quasi-brittle materials such as concrete lead to statistical distribution differences in the failure thresholds of each microelement. Weibull statistical damage theory assumes that the failure strain of micro-elements inside the material follows Weibull distribution, and its macroscopic damage variable D(ε) can be defined as follows:

(9) $$D(\varepsilon )=1-\exp\left[-\frac{\varepsilon^{m}}{\alpha }\right]$$

where α is the scale parameter of Weibull distribution, and m is the shape parameter of Weibull distribution. Using the Lemaitre equivalent stress principle, the macroscopic stress can be expressed as follows:

(10) $$\sigma_{D}(\varepsilon )=(1-D(\varepsilon ))\cdot \sigma (\varepsilon )$$

In the equation, $\sigma_{D}(\varepsilon )$ is the stress response of the material when it is not damaged, and by combining Equations (6) and (7), the fractional calculus constitutive relation considering high-temperature damage can be expressed as

(11) $$\sigma_{D}(\varepsilon )=\sigma (\varepsilon )(1-D(\varepsilon ))=\exp\left[-\frac{\varepsilon^{m}}{\alpha }\right]\frac{\xi }{\Gamma (2-\beta )}{\dot{\varepsilon }}^{\beta }\varepsilon^{\left(1-\beta \right)},\;(0\leq \beta \leq 1)$$

For the constitutive model described in Equation (11), the dimension of its parameter system is reduced by analytical mathematical transformation. Taking the differential extreme condition that the first derivative of the macroscopic stress with respect to the strain at the extreme point is 0 and the characteristic state condition that the macroscopic stress at the peak strain $\varepsilon_{pk}$ reaches the peak stress $\sigma_{pk}$ as the double constraints, the explicit expressions of the parameters α and m are analytically derived, namely:

(12) $$\left\{\begin{array}{l} \frac{d\sigma_{D}}{d\varepsilon }=Ce^{-\varepsilon^{m}/\alpha }\varepsilon^{-\beta }\left[(1-\beta )-\frac{m}{\alpha }\varepsilon^{m}\right]=0,\;C=\frac{\xi }{\Gamma (2-\beta )}{\dot{\varepsilon }}^{\beta }\\ \sigma_{pk}=C\varepsilon_{pk}^{1-\beta }\exp\left[-\frac{\varepsilon_{pk}^{m}}{\alpha }\right] \end{array}\right.$$

By solving the above formula, the expressions of α and m are obtained as follows:

(13) $$\alpha =\frac{m}{1-\beta }\varepsilon_{pk}^{m},\;m=\frac{1-\beta }{\ln\left(\frac{C\varepsilon_{pk}^{1-\beta }}{\sigma_{pk}}\right)}$$

Substituting Equation (13) into Equation (11) and combining with $\sigma_{pk}=DIF•\sigma_{pks}=(a+b\mathrm{lg}\dot{\varepsilon })\;\sigma_{pks}$, the fractional calculus constitutive model is obtained as follows:

(14) $$\sigma_{D}(\varepsilon ,\dot{\varepsilon })=\frac{\xi }{\Gamma (2-\beta )}{\dot{\varepsilon }}^{\beta }\varepsilon^{1-\beta }\cdot {\left(\frac{\xi {\dot{\varepsilon }}^{\beta }\varepsilon_{pk}^{1-\beta }}{\Gamma (2-\beta )\left(a+b\cdot \mathrm{lg}\dot{\varepsilon }\right)\sigma_{pks}}\right)}^{-\left(\frac{\varepsilon (1-\beta )}{\varepsilon_{pk}}/\ln\left(\frac{\xi {\dot{\varepsilon }}^{\beta }\varepsilon_{pk}^{1-\beta }}{\Gamma (2-\beta )\left(a+b\cdot \mathrm{lg}\dot{\varepsilon }\right)\sigma_{pks}}\right)\right)}$$

Part of Equation (14) is defined as follows:

(15) $$C=\frac{\xi }{\Gamma (2-\beta )}{\dot{\varepsilon }}^{\beta },\;\;A=\frac{\xi {\dot{\varepsilon }}^{\beta }\varepsilon_{pk}^{1-\beta }}{\Gamma (2-\beta )\sigma_{pk}}$$

Then the constitutive model of fractional calculus can be simplified as follows:

(16) $$\sigma_{D}(\varepsilon )=C\varepsilon^{1-\beta }\cdot A^{-{\left(\frac{\varepsilon }{\varepsilon_{pk}}\right)}^{\frac{1-\beta }{\ln A}}}$$

The constitutive relationship established by Equation (16) adopts the fractional calculus model instead of the complex Kelvin chain or Maxwell network in the traditional integer order model, and the unified description of elasticity, viscoelasticity, and viscosity is realized through parameters β. The modified equation contains five parameters: ξ, β, $\dot{\varepsilon }$, $\sigma_{pks}$, $\varepsilon_{pk}$, among which *ξ* and β can be determined by fitting the test data. This constitutive model breaks through the construction idea of the traditional viscoelastic constitutive model, which describes the instantaneous response with the elastic component and then describes the time dependence with the coupled viscous component. It also considers the influence parameters, such as temperature and strain rate, to regulate the weight of viscoelastic response, which ensures the applicability of the constitutive model to complex actual scenes.

**Figure 8:**
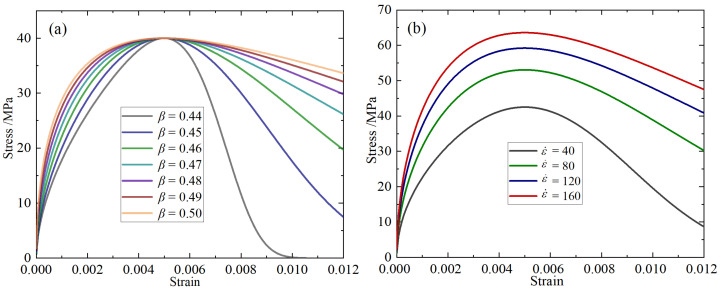
Constitutive parameter sensitivity analysis: (**a**) β; (**b**) $\dot{\varepsilon }$.

To clarify the influence of the key parameters in the constitutive model on the stress–strain response, a parameter sensitivity analysis was conducted on β and $\dot{\varepsilon }$, and the results are shown in Figure 8.

Figure 8a illustrates the influence law of parameter β on the curve shape. Under the conditions of ξ = 100 and $\dot{\varepsilon }$ = 80, when β increases from 0.44 to 0.50, the peak stress remains the same, but the curve shape significantly differs. The curve corresponding to a smaller β (such as 0.44) is steep overall, with the stress rapidly decaying after the peak, showing a strong brittle characteristic; while a larger β (such as 0.49 and 0.50) makes the curve more gentle, with the stress decreasing more slowly after the peak, demonstrating stronger ductility. It can be seen that β is the main controlling parameter determining the shape evolution of the softening segment and ductility of the material.

Figure 8b shows the influence law of $\dot{\varepsilon }$ on the stress–strain relationship. Under the conditions of β = 0.48 and ξ = 100, as $\dot{\varepsilon }$ increases from 40 to 160, the peak stress significantly increases, and the overall curve shows a significant upward trend. This indicates that this model can describe the strain rate strengthening effect of concrete well.

In conclusion, parameter β determines the curve shape and ductility, with $\dot{\varepsilon }$ reflecting the dynamic strengthening characteristics. Under the joint action of the two, the model can flexibly adapt to the concrete constitutive behavior under various loading conditions, verifying the scientificity and rationality of the structural design of the constitutive model in this paper.

### 4.2. Verification of the Correctness of the Constitutive Model

Taking the fiber content of 0.2% as an example, based on the test data under different temperatures and different strain rates, the constitutive parameters in Equation (16) were inversely calculated; the procedure was implemented in Python (version 3.10.6). The peak strain was automatically extracted from the experimental stress–strain data, and under the fixed conditions of peak strain and strain rate, the constitutive model parameters ξ and β were obtained by nonlinear least-squares fitting.

**Figure 9:**
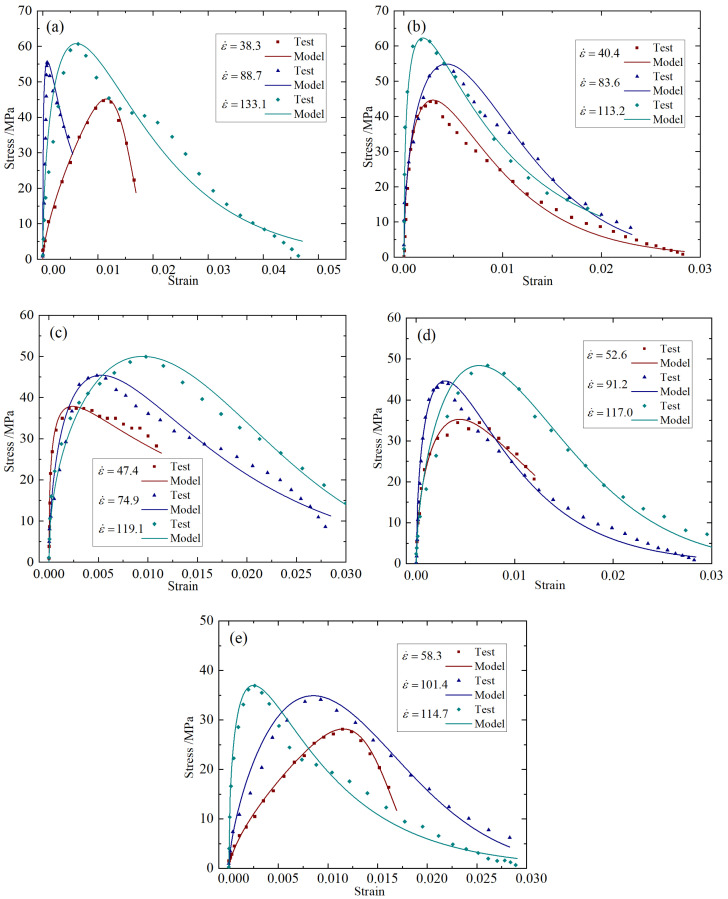
Constitutive model stress–strain curves: (**a**) 20 °C; (**b**) 200 °C; (**c**) 400 °C; (**d**) 600 °C; (**e**) 800 °C.

The specific parameters are presented in Table 4; the comparison between the theoretical values of the stress–strain curve and the experimental values is presented in Figure 9.

**Figure 10:**
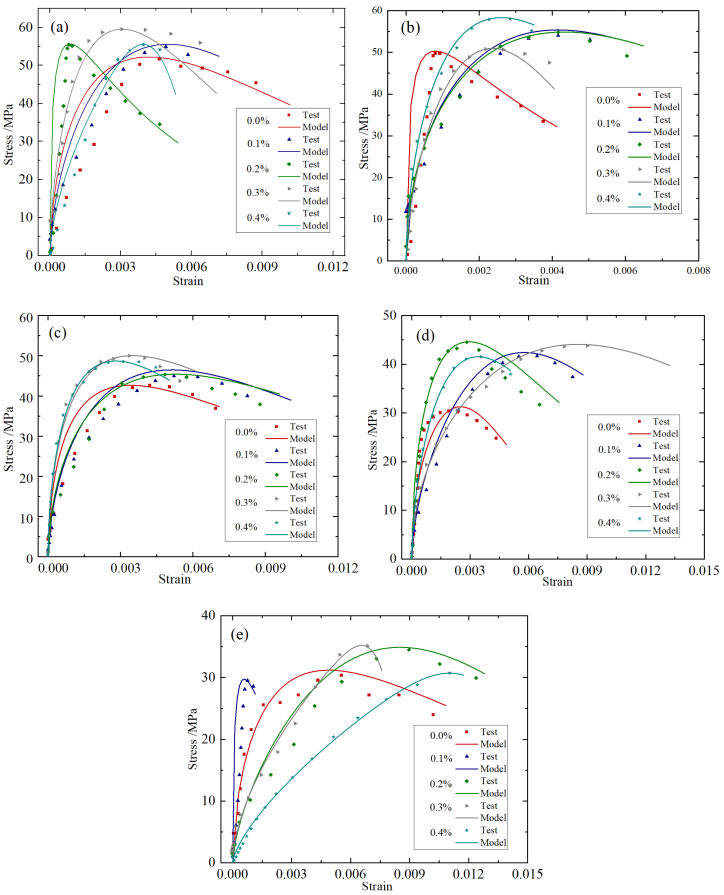
Constitutive model stress–strain curves: (**a**) 20 °C; (**b**) 200 °C; (**c**) 20 °C; (**d**) 200 °C; (**e**) 800 °C.

Taking the working condition of an impact speed of 8.8 m/s as an example, based on the test data under different temperatures and different fiber content conditions, the constitutive parameters in Equation (16) were inversely calculated. As shown in Table 4, the comparison of the theoretical values and the experimental values of the stress–strain curve is presented in Figure 10.

It can be seen from Figure 9 and Figure 10, and Table 4 and Table 5 that the theoretical curves of the constitutive model constructed in this paper are in good agreement with the experimental results, which can reflect the whole process of the dynamic compression failure of BFRC. The fitting degree R^2^ is always above 0.9, which verifies the correctness of the constitutive model in this paper. In addition, it can be seen from Table 4 and Table 5 that the value β of BFRC varies between 0.440 and 0.517 under various working conditions, and on the whole, the value β of BFRC increases with the increase in temperature, and the value β of BFRC is basically lower than that of plain concrete, which indicates that BFRC is a material with both elastic and viscous adaptability. Its viscoelastic properties are the result of coupled competition between elastic recovery and viscous flow.

In this paper, the constitutive model constructed based on the fractional calculus theory achieves a relatively accurate description of the dynamic viscoelastic mechanical response of BFRC with relatively simple expressions, which can describe the mechanical behavior of the transition state in the elastic–viscoelastic–viscous continuum of materials, and provide a theoretical basis for the quantitative analysis of the viscoelastic characteristics of BFRC materials.

## 5. Microscopic Mechanism Analysis of Viscoelastic Properties of BFRC

In order to further reveal the microscopic mechanism of the viscoelastic properties of BFRC after high temperature and water cooling, electron microscopy and nuclear magnetic resonance (NMR) tests were carried out by using a electron microscope scanner(Quanta 650, FEI, Hillsboro, OR, USA) and a Newmeyer nuclear magnetic resonance instrument(MesoMR23-060H-I, Niumag Corporation, Suzhou, China).

**Figure 11:**
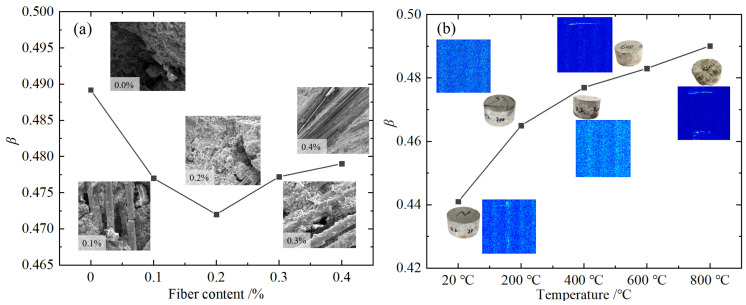
β parameter evolution curve: (**a**) different fiber content; (**b**) 0.2% BFRC after different temperatures.

For the conditions of 0.0–0.4% admixture, 20–800 °C temperature, and 8.8 m/s impact rate, the average value of each admixture at each temperature was taken as the value of the admixture, and the change law with fiber admixture was plotted as shown in Figure 11a. For the conditions of temperature 20–800 °C, impact rate 5.4 m/s, 8.8 m/s, 11.3 m/s, and fiber content 0.2%, the average value at each temperature and impact rate was taken as the value, and the variation law with temperature was drawn as shown in Figure 11b.

It can be seen from Figure 11a that the values of viscoelastic parameters β of BFRC are between 0.472 and 0.479, which are significantly lower than those of plain concrete. As a typical multiphase composite material, at the micro-scale level, the fiber improves the solid stress transfer mechanism through the bridge effect, and the viscous resistance of the fiber–matrix interface slips, so that the structure of the material is more compact under load, and the viscoelastic characteristics of the material tend to be elastic, while the internal structure of the specimen is loose when the fiber is not incorporated or there is too much fiber. The viscoelastic properties of BFRC tend to be viscous, which reflects the change in mechanical properties at the macroscopic scale. The viscoelastic properties of BFRC are essentially the cooperative response of its multiphase composite system at different scales.

It can be seen from Figure 11b that the viscoelastic property parameters β of BFRC increase with the increase in temperature, that is, the material viscoelastic properties of BFRC tend to develop from elastic to viscous after high temperature. On the one hand, combined with the pore distribution characteristics of NMR imaging under different temperature conditions, it can be seen that the higher the temperature, the more the pores in BFRC caused by water evaporation, thermal stress release, chemical change, and the phase transition of concrete. The increase in the number of pores will further lead to the loose internal structure of the concrete and reduce the rigidity. On the other hand, from the perspective of physical and chemical changes, the effect of high temperature will make the BFRC matrix produce a series of “softening” phenomena. Specifically, under high-temperature conditions, at 100–200 °C, the internal free water of concrete evaporates and tiny cracks appear [44], which destroys the rigid structure of the material. After 300 °C, the C-S-H gel of cement hydration products decomposes into compounds of lower strength and density [45]: $C_{1.62}SH_{1.5}\to [(1–\xi_{\mathrm{CSH}})C_{1.62}SH_{1.5}+0.62\xi_{\mathrm{CSH}}C_{2}S+0.38\xi_{\mathrm{CSH}}\mathrm{CS}]+1.5\xi_{\mathrm{CSH}}H↑$; at the same time, part of the chemically bound water will also evaporate with the decomposition of these hydration products, which will reduce the performance of the cement paste and interface transition zone and weaken the strengthening effect of fibers. After 400 °C, calcium hydroxide crystal decomposition occurs: $\mathrm{Ca}(\mathrm{OH}{)}_{2}\to \mathrm{CaO}+H_{2}O↑$; at 600 °C, limestone decomposes: $\mathrm{CaC}O_{3}\to \mathrm{CaO}+CO_{2}↑$ [15]. During the heating process, the internal structure of BFRC gradually becomes loose, the cohesion decreases continuously, and the material properties transition from elastic to viscous. The change mechanism of viscoelastic properties caused by high temperature has important reference value for optimizing the design and safe service operation of BFRC in a high-temperature environment.

In conclusion, BFRC has both elastic and viscous properties, and the appropriate amount of basalt fiber enhances its elastic characteristics by improving the stress transfer mechanism, and the microstructure damage caused by high temperature and a series of chemical changes strengthens its viscous characteristics.

## 6. Conclusions

(1) After exposure to high temperatures, the dynamic mechanical behavior of BFRC exhibits fiber-toughening effects, temperature-induced deterioration, and strain rate strengthening effects. The dynamic compressive strength decreases significantly with increasing temperature, increases markedly with higher impact rates, and shows a non-monotonic dependence on fiber content, reaching a maximum at a fiber volume fraction of approximately 0.2%. These findings are consistent with the results reported in previous studies [41,42,45].

(2) Based on the fractional calculus theory, the dynamic constitutive model of BFRC is constructed, which comprehensively considers the influence of temperature and strain rate on the viscoelastic response weights. Through the continuous adjustment of the fractional differential order, the mechanical behavior of the material in the transition state in the elastic–viscoelastic–viscous continuum can be described well. The construction method of the constitutive model in this paper breaks through the construction idea of the traditional viscoelastic constitutive model that uses the elastic component to describe the instantaneous response and the viscous component to describe the time dependence, and it has good applicability to complex actual scenes.

(3) BFRC has both elastic and viscous properties, and its viscoelastic characteristics are essentially the cooperative response of the multiphase composite system at different scales. Basalt fibers strengthen their elastic characteristics by improving the stress transfer mechanism, and high-temperature effects strengthen their viscous characteristics through high-temperature damage and deterioration of the microstructure and a series of chemical changes.

(4) The dynamic constitutive model of BFRC under high-temperature conditions proposed in this study effectively captures the material’s dynamic mechanical response and can be applied to performance evaluation and service life prediction of critical BFRC components subjected to elevated temperatures. Compared with previous studies on the post-fire constitutive behavior of concrete [3,13,14,15,16,17,18,19,20,21,22], the present model offers improved characterization of viscoelastic effects. Consequently, it provides both theoretical and numerical support for the design of fire- and impact-resistant structures, as well as for the assessment of their service safety. Future research may incorporate other fiber types and more complex loading scenarios to further explore the relationships between model parameters and additional influencing factors, thereby enhancing the model’s general applicability and predictive accuracy.

## Figures and Tables

**Table 1:** Basic material mix proportions.

Specimen	Cement	Sand	Stone	Water	Fly Ash	Water Reducer	Fiber
BFRC-0%	1.00	2.03	3.80	0.56	0.40	0.02	0
BFRC-0.1%	1.00	2.03	3.80	0.56	0.40	0.02	0.1
BFRC-0.2%	1.00	2.03	3.80	0.56	0.40	0.02	0.2
BFRC-0.3%	1.00	2.03	3.80	0.56	0.40	0.02	0.3
BFRC-0.4%	1.00	2.03	3.80	0.56	0.40	0.02	0.4

**Table 2:** Research plan information.

Specimen	Temperature	Impact Velocities	Replicates	Number of Specimens
BFRC-0%	20 °C	5.4 m/s, 8.8 m/s, 11.3 m/s	3	9
	200 °C	5.4 m/s, 8.8 m/s, 11.3 m/s	3	9
	400 °C	5.4 m/s, 8.8 m/s, 11.3 m/s	3	9
	600 °C	5.4 m/s, 8.8 m/s, 11.3 m/s	3	9
	800 °C	5.4 m/s, 8.8 m/s, 11.3 m/s	3	9
BFRC-0.1%	20 °C	5.4 m/s, 8.8 m/s, 11.3 m/s	3	9
	200 °C	5.4 m/s, 8.8 m/s, 11.3 m/s	3	9
	400 °C	5.4 m/s, 8.8 m/s, 11.3 m/s	3	9
	600 °C	5.4 m/s, 8.8 m/s, 11.3 m/s	3	9
	800 °C	5.4 m/s, 8.8 m/s, 11.3 m/s	3	9
BFRC-0.2%	20 °C	5.4 m/s, 8.8 m/s, 11.3 m/s	3	9
	200 °C	5.4 m/s, 8.8 m/s, 11.3 m/s	3	9
	400 °C	5.4 m/s, 8.8 m/s, 11.3 m/s	3	9
	600 °C	5.4 m/s, 8.8 m/s, 11.3 m/s	3	9
	800 °C	5.4 m/s, 8.8 m/s, 11.3 m/s	3	9
BFRC-0.3%	20 °C	5.4 m/s, 8.8 m/s, 11.3 m/s	3	9
	200 °C	5.4 m/s, 8.8 m/s, 11.3 m/s	3	9
	400 °C	5.4 m/s, 8.8 m/s, 11.3 m/s	3	9
	600 °C	5.4 m/s, 8.8 m/s, 11.3 m/s	3	9
	800 °C	5.4 m/s, 8.8 m/s, 11.3 m/s	3	9
BFRC-0.4%	20 °C	5.4 m/s, 8.8 m/s, 11.3 m/s	3	9
	200 °C	5.4 m/s, 8.8 m/s, 11.3 m/s	3	9
	400 °C	5.4 m/s, 8.8 m/s, 11.3 m/s	3	9
	600 °C	5.4 m/s, 8.8 m/s, 11.3 m/s	3	9
	800 °C	5.4 m/s, 8.8 m/s, 11.3 m/s	3	9

**Table 3:** Relevant parameters for DIF calculation.

Temperature	Static Strength (MPa)	Impact Velocities	Dynamic Strength (MPa)	Strain Rate (s^−1^)	DIF
20 °C	35.8	5.4 m/s	44.7	38.3	1.25
		8.8 m/s	55.6	88.7	1.55
		11.3 m/s	61.1	133.1	1.71
200 °C	36.4	5.4 m/s	45.4	40.4	1.25
		8.8 m/s	55.3	83.6	1.52
		11.3 m/s	62.7	113.8	1.72
400 °C	29.7	5.4 m/s	37.4	46.8	1.26
		8.8 m/s	45.9	75.2	1.55
		11.3 m/s	50.0	119.7	1.68
600 °C	27.6	5.4 m/s	34.7	52.6	1.26
		8.8 m/s	44.6	91.0	1.62
		11.3 m/s	48.2	117.4	1.75
800 °C	21.1	5.4 m/s	28.2	58.7	1.34
		8.8 m/s	36.1	101.2	1.71
		11.3 m/s	37.8	115.3	1.81

**Table 4:** Parameter values under different temperature and strain rate conditions.

Temperature (°C)	Strain Rate (s^−1^)	*ξ*	β	R^2^	*σ*_pks_ (MPa)
20	38.3	184	0.440	0.925	35.8
	88.7	235	0.466	0.963	
	133.1	179	0.417	0.973	
200	40.4	195	0.487	0.962	36.4
	83.6	73	0.456	0.939	
	113.2	180	0.452	0.947	
400	47.4	182	0.494	0.969	29.7
	74.9	193	0.472	0.925	
	119.1	75	0.465	0.942	
600	52.6	84	0.484	0.973	27.6
	91.2	197	0.492	0.903	
	117.0	150	0.473	0.913	
800	58.3	102.	0.483	0.947	21.1
	101.4	200	0.495	0.931	
	114.7	200	0.492	0.946	

**Table 5:** Partial operating condition constitutive model parameters.

Temperature (°C)	Fiber Dosage (%)	*ξ*	β	R^2^	*σ*_pks_ (MPa)
20	0.0	173	0.455	0.918	33.6
	0.1	149	0.451	0.932	34.5
	0.2	189	0.466	0.956	35.8
	0.3	215	0.467	0.896	39.3
	0.4	184	0.464	0.987	36.7
200	0.0	233	0.498	0.915	32.7
	0.1	188	0.450	0.964	35.4
	0.2	173	0.456	0.947	34.8
	0.3	178	0.464	0.948	33.0
	0.4	196	0.468	0.957	36.9
400	0.0	159	0.483	0.985	29.5
	0.1	165	0.478	0.921	30.2
	0.2	167	0.472	0.946	29.0
	0.3	157	0.480	0.917	33.8
	0.4	162	0.477	0.964	37.4
600	0.0	147	0.517	0.982	28.3
	0.1	106	0.494	0.971	27.1
	0.2	121	0.471	0.908	29.8
	0.3	129	0.483	0.963	27.5
	0.4	135	0.496	0.974	25.7
800	0.0	138	0.493	0.937	20.2
	0.1	181	0.512	0.916	17.6
	0.2	86	0.495	0.977	23.5
	0.3	76	0.492	0.958	22.0
	0.4	91	0.490	0.943	20.7

## Data Availability

The original contributions presented in this study are included in the article. Further inquiries can be directed to the corresponding author.
